# Asperosaponin VI Ameliorates Spontaneous Abortion by Inhibiting Trophoblast Ferroptosis via the KEAP1/NRF2/GPX4 Axis

**DOI:** 10.3390/antiox15060699

**Published:** 2026-05-31

**Authors:** Yangyang Duan, Xinyu Xiao, Jiahong Chen, Xianglun Ji, Jinghang Yang, Zhangrui Nie, Hairuo Chen, Yingxi Wei, Yuhan Wu, Zhonglin Chen, Fan Lin, Shu Jiang

**Affiliations:** College of Integrative Medicine, Fujian University of Traditional Chinese Medicine, Fuzhou 350122, China; dyy5528@163.com (Y.D.); xiaoxy03@163.com (X.X.); cjhoar123@163.com (J.C.); xl595n@163.com (X.J.); yjh1734643@163.com (J.Y.); n6561269@163.com (Z.N.); c280865@163.com (H.C.); wyx802316@163.com (Y.W.); wyh260026@163.com (Y.W.); czl29526@163.com (Z.C.)

**Keywords:** asperosaponin VI, HTR-8/SVneo, ferroptosis, spontaneous abortion, KEAP1/Nrf2/GPX4 axis

## Abstract

Spontaneous abortion (SA) is intimately associated with ferroptosis in placental trophoblasts. Asperosaponin VI (AVI), a major active triterpene saponin extracted from Dipsacus asperoides, has emerged as a promising therapeutic candidate for SA; however, its precise molecular mechanisms remain poorly elucidated. This study aimed to investigate the protective efficacy of AVI against SA and clarify its underlying pathways. In vivo, an SA model was established via the subcutaneous administration of bromocriptine. AVI intervention significantly reduced the embryo resorption rate and ameliorated placental injury. Biochemically, AVI attenuated the accumulation of MDA and Fe^2+^, downregulated pro-ferroptotic markers (TFR-1 and ACSL4), and systematically modulated the expression of SLC7A11 alongside the ferroptosis-related KEAP1/NRF2/GPX4 signaling axis. In silico analyses, including molecular docking and molecular dynamics simulations, confirmed a robust binding affinity between AVI and NRF2. In vitro, AVI dose-dependently reversed erastin-induced trophoblast dysfunction and mitochondrial impairment, while effectively abrogating the ferroptosis-associated overproduction of ROS, Fe^2+^, and MDA. Crucially, pharmacological inhibition of NRF2 by ML385 partially negated these cytoprotective effects. Comprehensive molecular analyses (RT-qPCR, IF and Western blotting) revealed that AVI suppressed KEAP1 while concomitantly upregulating NRF2, GPX4, and SLC7A11. In conclusion, AVI prevents SA by suppressing trophoblast ferroptosis through KEAP1/NRF2/GPX4 signaling, offering a novel therapeutic approach.

## 1. Introduction

Spontaneous abortion (SA), defined as the unintentional loss of pregnancy prior to 20 weeks of gestation, affects approximately 10–15% of clinically recognized pregnancies. Furthermore, recurrent pregnancy loss impacts an estimated 2.6% of women of reproductive age [1,2]. While the etiology of SA is multifactorial, encompassing immune dysregulation, genetic aberrations, environmental exposures, anatomical anomalies, and endocrine disorders, the underlying cause remains idiopathic in nearly 50% of cases [3,4]. Current clinical interventions primarily rely on hormonal supplementation and immunomodulatory agents [5,6]; however, their utility is frequently constrained by poorly defined mechanisms of action and burgeoning safety concerns [7]. Consequently, the development of novel therapeutic strategies for SA is urgently warranted.

The placenta serves as a critical interface sustaining intrauterine fetal development, with its structural and functional integrity directly dictating pregnancy outcomes. Trophoblasts, the primary functional units of the placenta, execute tightly regulated proliferation, differentiation, and invasion—processes indispensable for embryo implantation, placental angiogenesis, and the maintenance of gestation [8,9]. Emerging evidence indicates that trophoblast dysfunction constitutes a central pathological driver of various gestational complications. Specifically, impaired trophoblast invasion and aberrant differentiation disrupt proper placentation and vascular development. These defects trigger placental ischemia, oxidative stress, and compromised maternofetal exchange, all of which are inextricably linked to the pathogenesis of early spontaneous pregnancy loss [10,11]. Furthermore, aberrant trophoblast differentiation disrupts placental vasculogenesis and endocrine signaling, contributing to implantation failure and spontaneous pregnancy loss [12].

The placental trophoblast lineage primarily comprises three distinct subpopulations: cytotrophoblasts (CTBs), extravillous trophoblasts (EVTs), and syncytiotrophoblasts (STBs) [13]. Following blastocyst implantation, progenitor CTBs—located immediately adjacent to the embryo—either fuse to form the multinucleated STB layer, which interfaces directly with maternal blood, or differentiate into EVTs [14]. EVTs possess intrinsic invasive capacity, infiltrating the maternal uterine stroma and remodeling the vasculature to establish optimal maternofetal hemodynamics [15]. Consequently, impaired EVT migration and invasion are primary contributors to placental structural anomalies and early SA. This makes EVTs a critical cellular target for elucidating the pathogenesis of SA and evaluating potential pharmacological interventions.

Growing evidence underscores the pivotal role of ferroptosis in trophoblast dysfunction and the pathogenesis of SA. Ferroptosis is an iron-dependent, lipid peroxidation-driven modality of regulated cell death, primarily characterized by glutathione metabolic imbalance and the systemic accumulation of reactive oxygen species (ROS) [16,17]. Glutathione peroxidase 4 (GPX4) acts as a pivotal negative regulator of ferroptosis. Its diminished activity leads to the impaired clearance of lipid peroxides, consequently inducing trophoblast injury [18,19]. The Kelch-like ECH-associated protein 1 (KEAP1)/Nuclear factor erythroid 2-related factor 2 (NRF2)/GPX4 signaling axis constitutes a fundamental pathway governing cellular antioxidant defenses and ferroptotic sensitivity. Under hypoxic stress, activation of this axis in trophoblasts significantly upregulates the activities of antioxidant enzymes, including catalase (CAT), glutathione peroxidase (GSH-Px), and superoxide dismutase (SOD), thereby maintaining redox homeostasis [20]. In contrast, KEAP1 overexpression accelerates the ubiquitination and proteasomal degradation of NRF2, impairing its nuclear translocation and the subsequent transcription of GPX4, which ultimately promotes ferroptosis within placental tissues [21]. Consistent with these findings, clinical data indicate that in patients with SA, KEAP1 is significantly upregulated in trophoblasts, accompanied by the suppression of NRF2-regulated antioxidant genes and heightened oxidative stress [22]. Similarly, our previous studies have demonstrated that within the KEAP1/NRF2 signaling pathway, Taishan Panshi Powder activates NRF2 and thereby upregulates GPX4 expression to alleviate trophoblast injury [23]. Consequently, targeted modulation of the KEAP1/NRF2/GPX4 signaling pathway to inhibit trophoblast ferroptosis represents a compelling therapeutic strategy for the prevention and management of SA.

Asperosaponin VI (AVI) is a bioactive triterpenoid saponin derived from the roots of *Dipsacus asperoides*, a perennial medicinal herb belonging to the Caprifoliaceae family. This species is indigenous to the mountainous terrains of southwestern and central China (spanning the Sichuan, Hubei, Yunnan, and Guizhou provinces) and adjacent Asian territories [24]. AVI exhibits a broad spectrum of pharmacological properties, including anti-apoptotic, anti-inflammatory, and antioxidant activities [25]. Previous studies have demonstrated that AVI alleviates mitochondrial oxidative stress-induced injury and exerts potent cytoprotective effects on the placenta in SA models [26,27]. Emerging evidence further suggests that AVI may antagonize ferroptosis by modulating GPX4 expression [28]. However, the precise regulatory mechanisms through which AVI modulates trophoblast ferroptosis remain to be elucidated. The present study aims to determine whether AVI inhibits trophoblast ferroptosis via the KEAP1/NRF2/GPX4 signaling axis, thereby providing a mechanistic foundation for its potential clinical application in the prevention and management of SA.

## 2. Materials and Methods

### 2.1. Materials

Asperosaponin VI (AVI, C47H76O18, molecular weight: 929.10, ≥98%, Cat. No. B20204-20 mg) was purchased from Yuanye Bio-Technology (Shanghai, China); the NRF2 inhibitor ML385 (HY-100523) and ferroptosis inducer Erastin (HY-15763) were obtained from MedChemExpress (Monmouth Junction, NJ, USA); Bromocriptine mesylate (HJ20160030) was provided by Novartis AG (Basel, Switzerland). Primary antibodies used for Western blotting were as follows: Acyl-CoA Synthetase Long-chain family member 4 (ACSL4, 22401-1-AP), Transferrin Receptor 1 (TFR-1, 65236-1-Ig), KEAP1 (60027-1-Ig), NRF2 (80593-1-RR), GPX4 (66428-1-Ig), and β-actin (66009-1-Ig) were from Proteintech Group, Inc. (Rosemont, IL, USA); anti-Solute Carrier Family 7 Member 11 (SLC7A11, ab307601) was from Abmart Biotech (Shanghai, China); anti-SOD (PT0113R) was from ImmunoWay Biotechnology (Plano, TX, USA). Horseradish peroxidase (HRP)-conjugated secondary antibodies, including anti-rabbit IgG (RGAR001) and anti-mouse IgG(RGAM001), were from Proteintech Group, Inc. (Rosemont, IL, USA).

### 2.2. Animal

Specific pathogen-free (SPF) Sprague-Dawley (SD) rats (8 weeks old, 15 males and 15 females) were purchased from Shanghai SLAC Laboratory Animal Co., Ltd. (Shanghai, China). The animals were housed in a climate-controlled environment with a temperature of 24–26 °C, relative humidity of 40–70%, and a 12 h light/dark photoperiod, with free access to standard laboratory diet and water. All rats underwent a one-week acclimatization period prior to experimental initiation. Animal experimental protocols were reviewed and approved by the Ethics Committee of Fujian University of Traditional Chinese Medicine (Approval No.: FJTCMIACUC 2023152).

### 2.3. Animal Grouping and Treatment

Female and male SD rats were cohabitated at a 1:1 ratio, with the presence of a vaginal plug at 07:00 h the following morning designated as gestational day (GD) 0.5. Gravid rats were randomly allocated into three experimental cohorts (*n =* 5 per group): control, model, and AVI-treated groups. All animal procedures were conducted in strict accordance with the 3R principles (Replacement, Reduction, and Refinement). Based on preliminary embryo resorption rates (control: ~5%; model: ~70%; AVI: ~20%), the requisite sample size was determined using a power analysis for one-way ANOVA with a significance level (α) of 0.05 and a statistical power (1 − β) of 0.80. A sample size of five rats per group provided sufficient statistical power to detect significant differences while minimizing animal usage, thereby complying with institutional ethical mandates.

Rats in the control group received daily intragastric gavage of an equivalent volume of normal saline from GD 0.5 to GD 12.5. Rats in the model group were given daily subcutaneous injection of bromocriptine at 0.4 mg·kg^−1^·d^−1^ from GD 6.5 to GD 8.5 to induce SA. Rats in the AVI group received daily intragastric gavage of AVI at 60 mg·kg^−1^·d^−1^ from GD 0.5 to GD 12.5, combined with simultaneous subcutaneous injection of bromocriptine at 0.4 mg·kg^−1^·d^−1^ from GD 6.5 to GD 8.5. All rats were euthanized on GD 12.5, and placental tissues were harvested for subsequent assays.

Bromocriptine mesylate was dissolved in 75% ethanol to achieve a final concentration of 0.4 mg·mL^−1^. AVI was dissolved in normal saline to prepare a working solution with a concentration of 12 mg·mL^−1^. The intragastric administration volume of AVI was standardized to 5 mL·kg^−1^ of body weight for all treated animals.

### 2.4. Sample Collection and Tissue Processing

On GD 12.5, pregnant rats were anesthetized via intraperitoneal injection of 3% sodium pentobarbital (30 mg·kg^−1^), followed by abdominal aorta blood collection. The animals were then euthanized by cervical dislocation. The abdominal cavity was surgically opened to fully expose the uterus, which was bluntly dissected along the uterine horns, and placentas were meticulously detached from their implantation sites. Isolated placental tissues were thoroughly rinsed with phosphate-buffered saline (PBS) to remove residual blood. A portion of the tissues was snap-frozen and stored at −80 °C for biochemical assays, while another portion was fixed in 4% paraformaldehyde (PFA) for subsequent histological examinations.

### 2.5. Assessment of Embryo Resorption Rate

The embryo resorption rate was calculated using Equation (1):

(1) $$Embryo\;resorption\;rate\;\left(\%\right)=\;\frac{N_{resorbed}}{N_{viable}+N_{resorbed}}\;\times 100\%\;$$

where

N_resorbed_ = number of resorbed embryos;
N_viable_ = number of viable embryos.

Resorbed embryos were macroscopically identified by a significant reduction in gestational sac size, typically accompanied by evident hemorrhage or necrotic focal lesions within the placental units.

### 2.6. Hematoxylin and Eosin (H&E) Staining

Placental tissues fixed in 4% PFA for 24 h were dehydrated through a graded ethanol series, embedded in paraffin, and sectioned into 5 μm-thick slices. After baking, the sections were deparaffinized in xylene, rehydrated through descending grades of ethanol, and subjected to H&E staining with hematoxylin (Solarbio #G1140, Beijing, China) and eosin (Solarbio #G1100, Beijing, China) following the manufacturer’s protocols. Following staining, the tissue sections were dehydrated, cleared, and mounted using neutral balsam. Histopathological alterations across the decidua basalis, basal zone, and labyrinth zone were subsequently evaluated using a light microscope (Nikon, Tokyo, Japan). In accordance with the 2018 classification criteria for toxin-induced placental lesions in rats, specific attention was directed toward structural disorganization, edema, and necrosis within the labyrinth zone, alongside inflammatory cell infiltration in the decidua. These parameters were meticulously examined to assess the extent of placental damage and determine the therapeutic efficacy of the AVI intervention [29].

### 2.7. Molecular Docking

The three-dimensional (3D) structures of core target proteins (KEAP1, NRF2, GPX4, TFR-1, and ACSL4) were retrieved from the RCSB Protein Data Bank (https://www.rcsb.org (accessed on 2 December 2024)) in PDB format [30]. Semi-flexible molecular docking simulations were conducted using AutoDock Vina 1.2.0 (https://github.com/ccsb-scripps/AutoDock-Vina (accessed on 2 December 2024)) [31]. The obtained docking conformations were submitted to the CB-Dock2 server (https://cadd.labshare.cn/cb-dock2/ (accessed on 2 December 2024)) for re-docking, reliability verification, and visual analysis of binding interactions [32]. Visual inspection and interaction mapping of optimal docking poses were performed to evaluate the binding affinity and spatial complementarity between AVI and the target proteins.

### 2.8. Molecular Dynamics (MD) Simulation

To investigate the binding modes and thermodynamic stability of the AVI-KEAP1, AVI-NRF2, and AVI-GPX4 complexes, 100 ns MD simulations were performed using GROMACS 2022.6 (http://www.gromacs.org/ (accessed on 3 December 2024)) [33]. Protein and ligand structures were optimized with UCSF Chimera 1.16 (https://www.cgl.ucsf.edu/chimera/ (accessed on 3 December 2024)) and ChemBio 3D 20.0 (http://www.chemdraw.com.cn/ (accessed on 3 December 2024)), respectively. System topology files were generated via the CGENFF server (https://cgenff.com (accessed on 3 December 2024)), and the CHARMM36 all-atom force field (https://academiccharmm.org/ (accessed on 3 December 2024)) was used for parameterization. Following energy minimization and NVT/NPT equilibration, the 100 ns production simulation was conducted at a constant temperature of 300 K and pressure of 1 bar [34]. Trajectory analyses included assessments of root mean square deviation (RMSD), root mean square fluctuation (RMSF), radius of gyration (Rg), and hydrogen bond dynamics, with results visualized using the DuIvyTools 0.4.6 package (https://pypi.org/project/DuIvyTools/ (accessed on 3 December 2024)).

### 2.9. Binding Free Energy Calculation

The binding free energy of the protein-ligand complexes was calculated using the gmx_MMPBSA tool 1.6.5 based on the single-trajectory approximation and molecular mechanics/Poisson-Boltzmann surface area (MM/PBSA) method.

### 2.10. Cell Culture and Experimental Grouping

The human extravillous trophoblast cell line (HTR-8/SVneo), an immortalized EVT-like model, was purchased from Immocell (IM-H525, Wuhan, China) and maintained in RPMI-1640 medium (Biosharp #BL303A, Hefei, China) supplemented with 10% fetal bovine serum (Immocell #IMC-105-50, Wuhan, China), 100 U·mL^−1^ penicillin, and 100 μg·mL^−1^ streptomycin. Cells were cultured in a humidified incubator at 37 °C under a 5% CO_2_ atmosphere [35].

For in vitro investigations, HTR/SVneo cells were divided into six experimental groups: Control group, Erastin group, AVI groups (50, 100, and 200 μmol·L^−1^), and ML385 group. All groups except the Control group were exposed to 20 μmol·L^−1^ Erastin to induce ferroptosis. AVI groups were co-treated with corresponding concentrations of AVI, while the ML385 group received combined treatment with 200 μmol·L^−1^ AVI and 15 μmol·L^−1^ ML385. All cells were cultured for 24 h prior to downstream assays.

### 2.11. Cell Viability Assay

Cell viability of HTR-8/SVneo cells was assessed using the Cell Counting Kit-8 (CCK-8). Briefly, cells were seeded in 96-well plates at a density of 1 × 10^4^ cells per well and cultured for 24 h to allow attachment. After treatment as described in the experimental grouping, the original medium was discarded, and each well was supplemented with 10 μL CCK-8 reagent (Beyotime #C0038, Shanghai, China) and 90 μL RPMI-1640 medium. Following incubation at 37 °C for 2 h in the dark, the absorbance was measured at 450 nm using a multi-mode microplate reader.

### 2.12. Wound Healing Assay

Cells were seeded in 6-well plates and cultured until reaching >90% confluence. A uniform linear scratch wound was created by vertically scraping the cell monolayer with a sterile 200 μL pipette tip. The wells were gently rinsed twice with PBS to remove cellular debris and detached cells, followed by treatment as described in the experimental grouping. Representative images of the same visual fields were captured under an inverted microscope (Nikon, Japan) immediately after wounding (0 h) and 24 h post-wounding. The residual scratch area was quantified using Image J 1.51 software (National Institutes of Health, USA), and cell migration capacity was expressed as the wound closure percentage. The cell migration rate was calculated using Equation (2):

(2) $$Cell\;migration\;rate\;\left(\%\right)=\;\frac{S_{0}-S_{24}}{S_{0}}\;\times 100\%\;$$

where

S_0_ = Scratch area at 0 h;
S_24_ = Scratch area at 24 h.

### 2.13. Measurement of Mitochondrial Membrane Potential

Cells in the logarithmic growth phase were seeded in 6-well plates and treated as outlined in the experimental grouping. After removing the culture supernatant, the cell monolayers were rinsed three times with PBS. Each well was supplemented with 1 mL of JC-1 staining working solution, followed by incubation at 37 °C for 20 min in the dark. Post-incubation, the cells were thoroughly rinsed three times with JC-1 staining buffer to remove excess dye, and fresh culture medium was added. Mitochondrial membrane potential was evaluated by capturing representative images using a fluorescence microscope (Nikon, Japan).

### 2.14. Transmission Electron Microscopy (TEM)

After treatment as outlined in the experimental grouping, HTR-8/SVneo cells were harvested and fixed in 2.5% glutaraldehyde (Servicebio #G1102, Wuhan, China) at 4 °C for 2 h. The samples were subjected to graded ethanol dehydration, transitioned with propylene oxide (twice for 10 min each), progressively infiltrated with EPON 812 resin, and polymerized at 60 °C for 24 h. Following block trimming, 70 nm ultrathin sections were cut using an ultramicrotome, double-stained with 2% uranyl acetate and 2.6% lead citrate (5 min each), gently rinsed with deionized water, and air-dried. Ultrastructural alterations, particularly in mitochondrial morphology, were visualized and imaged using a transmission electron microscope (HT7700 TEM, Hitachi, Tokyo, Japan).

### 2.15. Quantification of Intracellular Fe^2+^ and MDA Levels

After treatment as outlined in the experimental grouping, intracellular ferrous iron (Fe^2+^) and malondialdehyde (MDA) levels were quantified using the Cell Ferrous Ion Colorimetric Assay Kit (Jonlnbio #JL-T1255-96, Shanghai, China) and Cell MDA Colorimetric Assay Kit (Jonlnbio #JL-T1116-96, Shanghai, China), respectively, in strict accordance with the manufacturers’ protocols.

### 2.16. Assessment of Intracellular ROS

Intracellular ROS levels were evaluated using the 2′,7′-dichlorofluorescein diacetate (DCFH-DA) fluorescent probe (Elabscience #E-BC-K138-F, Wuhan, China). After treatment as outlined in the experimental grouping, the culture medium was discarded and replaced with serum-free medium containing 10 μmol·L^−1^ DCFH-DA. The cells were incubated at 37 °C for 45 min in the dark to allow deacetylation of DCFH-DA to DCFH and its subsequent oxidation to the fluorescent 2′,7′-dichlorofluorescein (DCF). Subsequently, the cells were rinsed three times with pre-warmed serum-free medium to remove extracellular probe. Representative images were captured using a fluorescence microscope, and the mean fluorescence intensity (MFI) of each field was quantified using Image J 1.51 software to reflect relative ROS accumulation.

### 2.17. Immunofluorescence (IF) Analysis

HTR-8/SVneo cells seeded in 6-well plates were treated as outlined in the experimental grouping, then fixed with 4% PFA and permeabilized with 0.1% Triton X-100. Non-specific binding was blocked with 5% goat serum at room temperature, after which the cells were incubated overnight at 4 °C with primary antibodies against KEAP1, NRF2 and GPX4 (all diluted 1:500). Following PBS rinsing, the cells were incubated with fluorophore-conjugated secondary antibodies at room temperature in the dark, and nuclei were counterstained with DAPI. Fluorescence images were captured using a fluorescence microscope to assess the subcellular localization and expression of the target proteins.

### 2.18. Reverse Transcription-Quantitative Polymerase Chain Reaction (RT-qPCR)

Total RNA was extracted from HTR-8/SVneo cells using TRIzol reagent (Vazyme #RC202-01, Nanjing, China) according to the manufacturer’s instructions. The concentration and purity of the isolated RNA were determined using a NanoDrop spectrophotometer (Gene Company Limited, Hong Kong, China). Subsequently, first-strand cDNA was synthesized using the SPARKscript II All-in-one RT SuperMix for qPCR Kit (Sparkjade #AG0305-B, Jinan, China). The primer sequences used in this study are provided in Table 1. Quantitative PCR was performed on a Bio-Rad 96 FEX system (Bio-Rad, Hercules, CA, USA) using 2× SYBR Green qPCR Mix (Sparkjade #AH0104-B, Jinan, China). Each 20 μL reaction mixture contained 10 μL of 2× SYBR Mix, 1 μL of cDNA template, 0.4 μL each of forward and reverse primers, and 8.2 μL of nuclease-free water. The thermal cycling conditions consisted of an initial denaturation at 95 °C for 30 s, followed by 40 cycles of denaturation at 95 °C for 15 s and annealing/extension at 60 °C for 30 s. Relative gene expression was expressed as the ratio of target gene to reference gene, with β-Actin serving as the internal reference gene for normalization.

### 2.19. Western Blotting Analysis

Total protein was extracted from placental tissues and HTR-8/SVneo cells using awestern & IP lysis buffer supplemented with a 1% protease inhibitor cocktail (Beyotime #P0013/P1005, Shanghai, China). Protein concentrations were quantified using a bicinchoninic acid (BCA) protein assay kit (Boster #AR1189, Wuhan, China). The protein samples were mixed with 5 times loading buffer (Beyotime #P0015, Shanghai, China) and denatured at 100 °C for 15 min. Equivalent amounts of protein (30 µg per lane) were separated via SDS/PAGE (Epizyme #PG112/PG113, Shanghai, China) and subsequently transferred onto polyvinylidene fluoride (PVDF) membranes (Sigma-Aldrich #ISEQ00010, St. Louis, MO, USA). To prevent non-specific binding, the membranes were blocked with a rapid blocking buffer (Beyotime #P0023B, Shanghai, China) at room temperature for 25 min. The membranes were then incubated overnight at 4 °C with primary antibodies targeting ACSL4, TFR-1, KEAP1, NRF2, GPX4, SLC7A11, SOD, and β-actin (all diluted at 1:5000). Following three washes with TBST, the membranes were incubated with HRP-conjugated secondary antibodies (anti-rabbit or anti-mouse IgG) at room temperature for 1 h. Protein bands were visualized using an ECL reagent and captured with a multifunctional imaging system. Densitometric analysis was performed using Image Lab 6.1 software (Bio-Rad, Hercules, CA, USA), and the expression levels of target proteins were normalized to that of β-actin as the internal loading control.

### 2.20. Statistical Analysis

All data are expressed as the mean ± standard deviation (SD). Statistical analyses were performed using SPSS 27.0 and GraphPad Prism 10.1.2 software. Comparisons among multiple groups were conducted using ANOVA, followed by the LSD post hoc test for equal variances or the Games-Howell test for unequal variances. A value of *p* < 0.05 was considered statistically significant.

## 3. Results

### 3.1. AVI Alleviates Embryo Resorption in SD Rats with SA via the KEAP1/NRF2/GPX4 Axis

**Figure 1:**
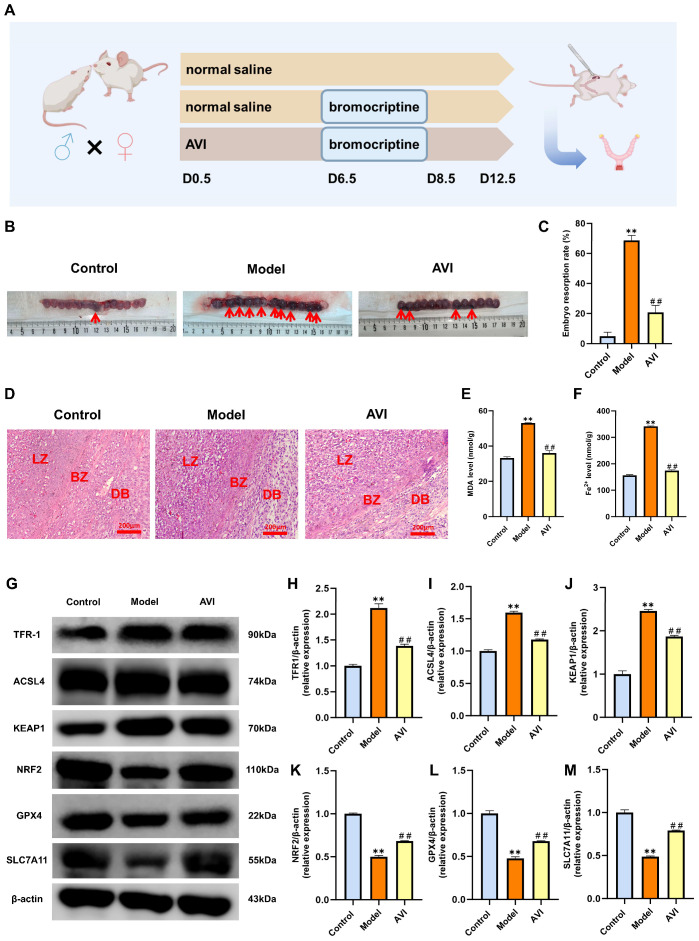
AVI reduces the embryo resorption rate in SD rats via the KEAP1/NRF2/GPX4 axis. (**A**) Flow chart of the pregnant rat modeling protocol. Rats in the Model group received subcutaneous bromocriptine injections from gestational days (GD) 6.5 to 8.5; Rats in the AVI group were intragastrically administered AVI from GD 0.5 to 12.5 alongside subcutaneous bromocriptine from GD 6.5 to 8.5. (**B**) Representative images of external embryonic morphology (arrows indicate congestion and resorption sites). (**C**) Embryo resorption rates (*n =* 5). (**D**) H&E staining of placental sections (LZ: labyrinth zone; BZ: basal zone; DB: decidua basalis; scale bar: 200 μm). (**E**,**F**) Placental levels of MDA and Fe^2+^ (*n =* 3). (**G**) Western blotting analysis of ferroptosis-related proteins in placental tissues, with β-actin serving as the loading control. (**H**–**M**) Densitometric quantification of TFR-1, ACSL4, KEAP1, NRF2, GPX4, and SLC7A11 protein expression (*n =* 3). Data are presented as mean ± SD. ** *p* < 0.01 vs. Control group; ^##^ *p* < 0.01 vs. Model group.

The experimental paradigm is illustrated in Figure 1A. To evaluate the efficacy of AVI on pregnancy outcomes in a bromocriptine-induced rat model of SA, we assessed overall embryonic morphology and resorption rates. As depicted in Figure 1B, embryos in the control group exhibited normal developmental morphology, characterized by pale pink coloration, intact fetal membranes, and distinct anatomical contours. In contrast, embryos from the model group showed dark purple discoloration indicative of congestion, focal resorption, and marked size heterogeneity, thereby confirming successful establishment of the bromocriptine-induced SA model. AVI treatment substantially ameliorated these gross pathological changes, mitigating congestion, restoring normal morphological integrity, and significantly reducing the incidence of embryonic resorption (Figure 1B,C). These macroscopic findings demonstrate that AVI effectively mitigates embryo resorption and improves pregnancy outcomes in the SA rat model.

Histological evaluation via H&E staining revealed region-specific placental histopathological alterations. Placentas from the control group demonstrated distinct stratification of the decidua basalis, basal zone, and labyrinth zone. The labyrinth zone displayed a compact, well-organized spongioform architecture with regular maternofetal vascular spaces, whereas the decidual and junctional zones maintained typical cellular morphology devoid of overt inflammation or necrosis. In the model group, the labyrinth zone sustained the most severe damage, characterized by structural disorganization, indistinct vascular margins, diminished vascular density, and prominent trophoblast necrosis. Concurrently, the decidual zone exhibited marked inflammatory cell infiltration and prominent edema, while the junctional zone presented substantial cellular disarray and a loss of normal trophoblast organization. Notably, AVI administration significantly mitigated these pathological aberrations. Following treatment, the labyrinth zone regained a highly ordered structure with substantially reduced necrosis and vacuolization. Furthermore, decidual inflammatory infiltration was markedly diminished, and the junctional zone demonstrated recovered cellular arrangement. These results suggest that AVI may contribute to the recovery of structural integrity in various functional regions of the placenta in SA model rats (Figure 1D).

To delineate the underlying molecular mechanisms of AVI, we quantified markers of oxidative stress and ferroptosis in the placental tissues. Levels of MDA and Fe^2+^ were significantly elevated in the model group; however, AVI treatment markedly abrogated these increases, suggesting an attenuation of placental oxidative damage (Figure 1E,F). Subsequently, the expression profiles of proteins associated with the KEAP1/NRF2/GPX4 signaling pathway and ferroptosis were assessed via Western blotting analysis. In the model group, the expression levels of TFR-1, ACSL4, and KEAP1 were significantly upregulated, whereas NRF2, GPX4, and SLC7A11 were correspondingly downregulated. Crucially, AVI intervention significantly reversed these aberrant expression patterns (Figure 1G–M). Collectively, these findings demonstrate that AVI exerts a robust cytoprotective effect on placental tissue in SA rats by modulating the KEAP1/NRF2/GPX4 signaling axis and regulating ferroptosis-associated proteins.

### 3.2. Molecular Docking

**Figure 2:**
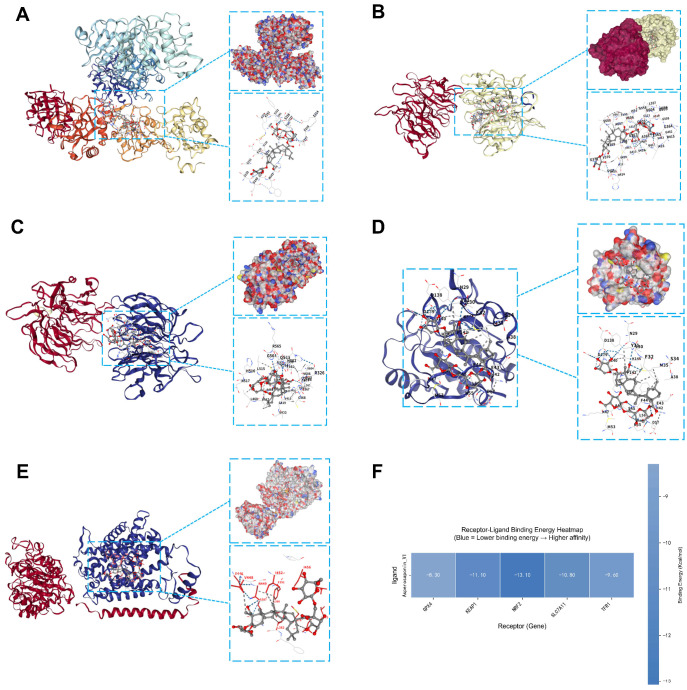
Molecular docking analysis of AVI and target proteins. (**A**–**E**) Optimal binding conformations of AVI with TFR-1, KEAP1, NRF2, GPX4, and SLC7A11. Dashed lines denote intermolecular interactions with surrounding amino acid residues: strong hydrogen bonds (dark blue), weak hydrogen bonds (light blue), ionic interactions (yellow), hydrophobic interactions (gray), Hydrogen Bond (red) and Cation-Pi Interaction (orange). (**F**) Heat map of receptor-ligand binding energies, where darker blue signifies lower binding energy and higher affinity.

Molecular docking was performed to evaluate the binding modes and interaction affinities between AVI and key pathway-related proteins. Our results demonstrate that AVI interacts with TFR-1 by forming hydrogen bonds with residues NAG2 and GLN347, yielding a binding free energy of −9.60 kcal/mol (Figure 2A). Similarly, AVI established hydrogen bonds with VAL514 and VAL561 of KEAP1 (−11.10 kcal/mol; Figure 2B); ARG470 and VAL514 of NRF2 (−13.10 kcal/mol; Figure 2C); PRO110 and ASP107 of GPX4 (−8.30 kcal/mol; Figure 2D); and ASN382, PRO488, and GLY299 of SLC7A11 (−10.80 kcal/mol; Figure 2E). Overall, docking analyses revealed robust hydrogen bonding and hydrophobic interactions between AVI and these critical ferroptosis-related proteins (TFR-1, KEAP1, NRF2, GPX4 and SLC7A11). The calculated binding free energies consistently indicated high binding affinities across all targets. Notably, the binding affinity between AVI and the DRM domain of NRF2 was markedly superior to that of the other examined proteins (Figure 2F).

### 3.3. MD Simulation

**Figure 3:**
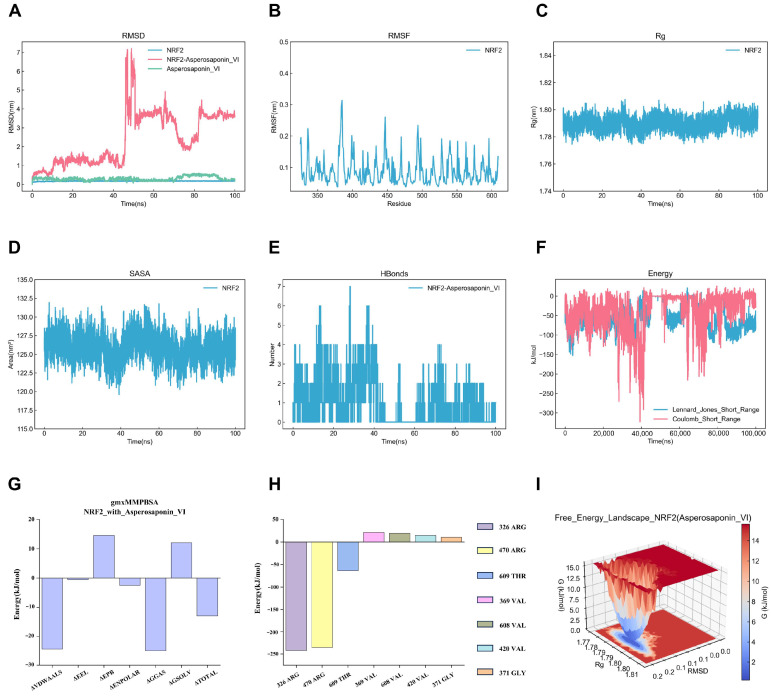
MD simulation of the NRF2-AVI complex. (**A**) RMSD trajectories of the NRF2 apoprotein, the NRF2-AVI complex, and the AVI ligand. (**B**) RMSF of the complex. (**C**) Rg of the complex. (**D**) SASA of the complex over time. (**E**) Number of intermolecular hydrogen bonds formed during the simulation. (**F**) Van der Waals and short-range electrostatic interaction energies. (**G**) Energy components of the binding free energy calculated via the MM/PBSA method. (**H**) Per-residue free energy decomposition highlighting key amino acid contributions. (**I**) FEL of the complex.

To further assess the dynamic binding stability of the AVI-protein complexes, MD simulations were conducted. RMSD analysis revealed that the NRF2-AVI complex remained highly stable, exhibiting minimal deviation (<0.2 nm) and reaching a steady-state equilibrium after 85 ns (Figure 3A). Throughout the simulation, the overall RMSF of the protein remained low, albeit with pronounced localized fluctuations near residues 370 and 450, suggesting these may serve as primary ligand-binding sites (Figure 3B). Assessment of structural compactness and solvent exposure demonstrated that the Rg remained relatively constant (Figure 3C), while the SASA plateaued after 85 ns (Figure 3D), indicating enhanced structural stabilization of the protein following ligand binding. Hydrogen bond trajectory analysis showed the transient formation of 2 to 4 hydrogen bonds during the initial 0–40 ns, which stabilized to a consistent 1 to 3 bonds between 65 and 100 ns (Figure 3E). Energy decomposition revealed that van der Waals forces were the predominant thermodynamic drivers during the terminal 85–100 ns phase, facilitating a stable complex between AVI and NRF2 (Figure 3F). Furthermore, MM/PBSA analysis yielded a binding free energy of −12.31 kJ/mol (Figure 3G), corroborating the stable interaction between the protein and the ligand. Per-residue free energy decomposition identified ARG326, ARG470, and THR609 as key contributors to the binding affinity, aligning closely with the observed RMSF profiles (Figure 3H). Finally, the free energy landscape (FEL) displayed a single energetic minimum (Figure 3I), confirming that the protein-ligand complex maintained a single, stable conformational state. Collectively, these data provide compelling evidence that AVI forms a highly stable complex with the NRF2 protein.

**Figure A1:**
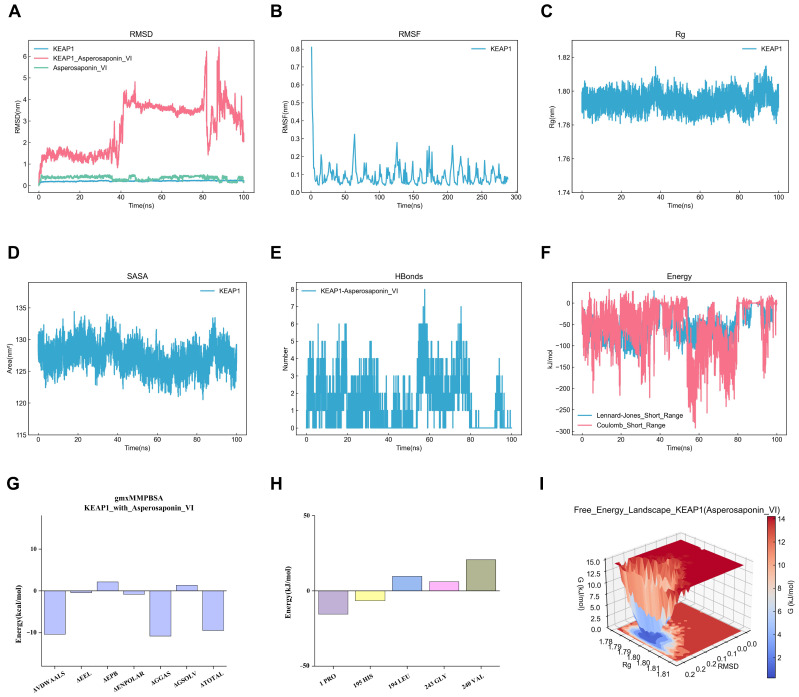
MD simulation of the KEAP1-AVI complex. (**A**) RMSD trajectories for the KEAP1 apoprotein, the KEAP1-AVI complex, and the AVI ligand. (**B**) RMSF profile of the complex. (**C**) Rg of the complex. (**D**) SASA plots over the simulation timescale. (**E**) Number of intermolecular hydrogen bonds formed. (**F**) Van der Waals and short-range electrostatic interaction energies. (**G**) Energy components of the MM/PBSA binding free energy. (**H**) Per-residue free energy decomposition indicating individual amino acid contributions. (**I**) FEL of the KEAP1-AVI complex.

**Figure A2:**
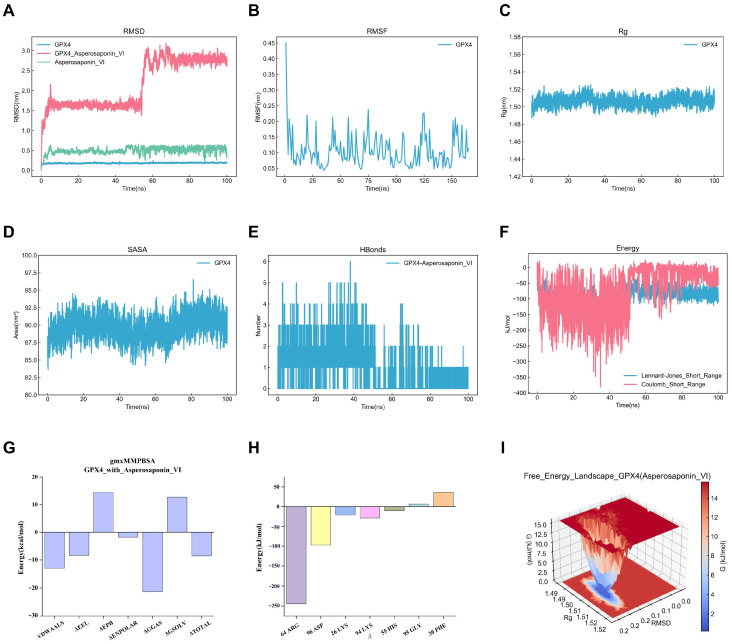
MD simulation of the GPX4-AVI complex. (**A**) RMSD trajectories for the GPX4 apoprotein, the GPX4-AVI complex, and the AVI ligand. (**B**) RMSF profile of the complex. (**C**) Rg of the complex. (**D**) SASA plots over the simulation timescale. (**E**) Number of intermolecular hydrogen bonds formed. (**F**) Van der Waals and short-range electrostatic interaction energies. (**G**) Energy components of the MM/PBSA binding free energy. (**H**) Per-residue free energy decomposition indicating individual amino acid contributions. (**I**) FEL of the GPX4-AVI complex.

As summarized in Table 2, Figure A1 and Figure A2, while AVI exhibited measurable binding affinities for both KEAP1 (−9.47 kJ/mol) and GPX4 (−8.57 kJ/mol), its interaction with NRF2 demonstrated the greatest thermodynamic favorability, yielding a binding free energy of −12.31 kJ/mol. MD simulations further corroborated these energetic profiles. In contrast to the transient AVI-KEAP1 complex, the AVI-NRF2 and AVI-GPX4 complexes achieved robust conformational stability during the terminal phase of the simulations (after 85 ns and 75 ns, respectively). These stable conformations were predominantly driven by van der Waals forces, with the structural integrity of the AVI-NRF2 complex being additionally reinforced by one to three persistent hydrogen bonds. Collectively, these computational findings indicate that AVI likely modulates this signaling pathway via NRF2. To empirically validate these pharmacological effects and further elucidate the underlying intervention mechanism, subsequent in vitro experiments will be conducted utilizing the specific NRF2 inhibitor ML385.

### 3.4. AVI Alleviates Erastin-Induced Ferroptosis in HTR-8/SVneo Cells via NRF2 Regulation

**Figure 4:**
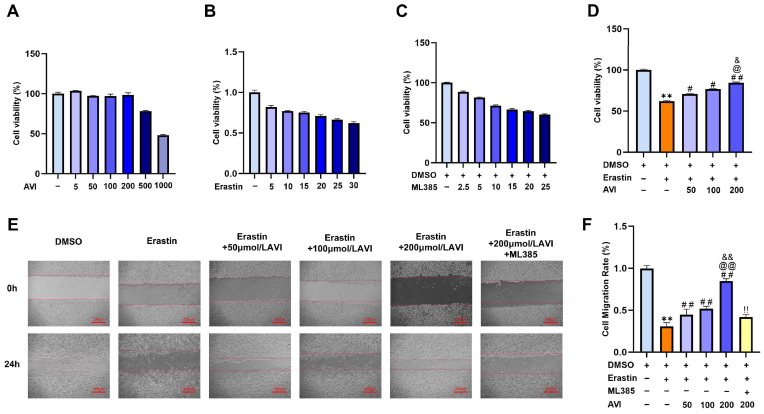
AVI mitigates Erastin-induced cytotoxicity and preserves migration in HTR-8/SVneo cells. HTR-8/SVneo cells were exposed to 20 μmol·L^−1^ Erastin for 24 h. The AVI groups were subsequently treated with 50, 100, or 200 μmol·L^−1^ AVI, while the ML385 group received 15 μmol·L^−1^ ML385 alongside 200 μmol·L^−1^ AVI, all for an additional 24 h. (**A**–**C**) Effects of varying concentrations of AVI, Erastin, and ML385 on cell proliferation, respectively. (**D**) Cell viability assessed via CCK-8 assay across different treatment conditions. (**E**,**F**) Cell migration capacity evaluated by wound healing assay. Data are presented as mean ± SD (CCK-8 assay: *n =* 5; Wound healing assay: *n =* 3). ** *p* < 0.01 vs. Control group; ^#^ *p* < 0.05, ^##^ *p* < 0.01 vs. Model group; ^@^ *p* < 0.05, ^@@^ *p* < 0.01 vs. 50 μmol·L^−1^ AVI group; ^&^ *p* < 0.05, ^&&^ *p* < 0.01 vs. 100 μmol·L^−1^ AVI group; ^!!^ *p* < 0.01 vs. 200 μmol·L^−1^ AVI group.

We first utilized a CCK-8 assay to evaluate the impact of varying AVI concentrations on HTR-8/SVneo cells viability. Following a 24-h incubation, AVI concentrations up to 200 μmol·L^−1^ exerted no significant inhibitory effect on cellular proliferation (Figure 4A). Consequently, we selected 50, 100, and 200 μmol·L^−1^ AVI for subsequent intervention experiments. Additionally, treatment with 20 μmol·L^−1^ Erastin or 15 μmol·L^−1^ ML385 significantly reduced cell viability; thus, these baseline concentrations were utilized in downstream assays (Figure 4B,C).

Further CCK-8 analysis revealed that Erastin treatment markedly suppressed cell viability, whereas AVI co-treatment rescued this damage in a dose-dependent manner (Figure 4D). Notably, co-administration of the NRF2 inhibitor ML385 significantly blunted the cytoprotective effect of AVI. Wound healing assays demonstrated that Erastin drastically impaired cell migration relative to the control. AVI ameliorated this Erastin-induced migratory inhibition dose-dependently, an effect that was largely abrogated by ML385 (Figure 4E,F). Collectively, these findings suggest that AVI mitigates Erastin-induced impairments in HTR-8/SVneo cells viability and migration via NRF2 activation.

**Figure 5:**
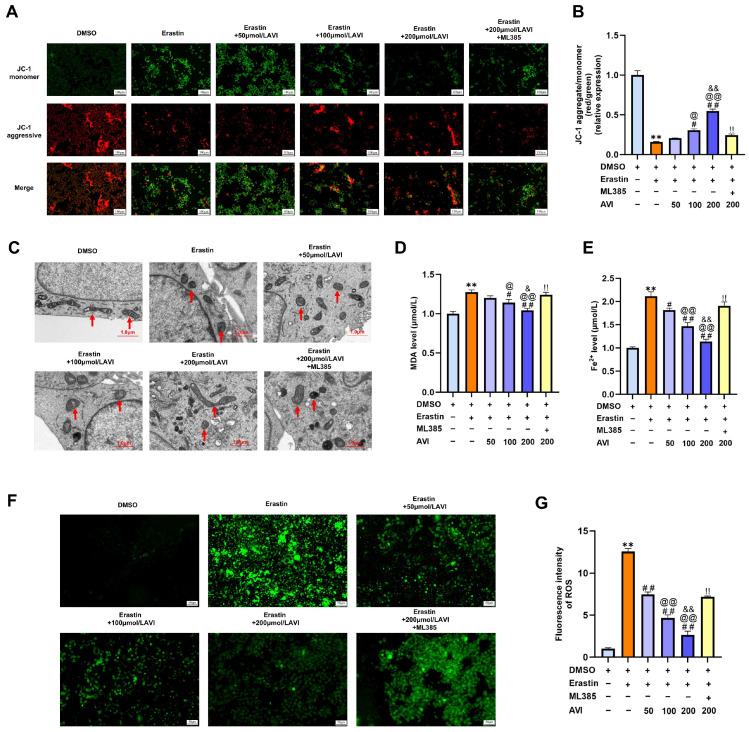
AVI protects HTR-8/SVneo cells by inhibiting ferroptosis. (**A**,**B**) Assessment of mitochondrial membrane potential. Scale bar: 100 μm. (**C**) TEM images detailing ferroptosis-induced mitochondrial damage. (Arrows indicate mitochondria in each group. Mitochondrial shrinkage, increased membrane electron density, reduced cristae and ruptured outer membrane are typical morphological characteristics of ferroptosis. Scale bar: 1 μm). (**D**,**E**) Intracellular levels of MDA and Fe^2+^. (**F**,**G**) Intracellular ROS accumulation detected via fluorescent probes. Scale bar: 50 μm. Data are presented as mean ± SD (*n =* 3). ** *p* < 0.01 vs. Control group; ^#^ *p* < 0.05, ^##^ *p* < 0.01 vs. Model group; ^@^ *p* < 0.05, ^@@^ *p* < 0.01 vs. 50 μmol·L^−1^ AVI group; ^&^ *p* < 0.05, ^&&^ *p* < 0.01 vs. 100 μmol·L^−1^ AVI group; ^!!^ *p* < 0.01 vs. 200 μmol·L^−1^ AVI group.

To ascertain whether AVI suppresses ferroptosis via NRF2 signaling, we systematically evaluated cellular ultrastructure, mitochondrial function, and relevant molecular markers. JC-1 staining revealed that Erastin drastically diminished the mitochondrial membrane potential (MMP) in HTR-8/SVneo cells. AVI effectively restored the MMP in a dose-dependent manner; however, this restoration was significantly attenuated by ML385 (Figure 5A,B). TEM demonstrated that Erastin induced classic ferroptotic morphological changes, including mitochondrial shrinkage, increased membrane density, and diminished cristae. AVI intervention reversed these ultrastructural abnormalities across all tested concentrations, whereas the addition of ML385 compromised this protection (Figure 5C). Furthermore, assessing oxidative stress and iron metabolism indices showed that Erastin induced a significant accumulation of intracellular Fe^2+^, MDA, and ROS. AVI efficiently reversed these elevations, but its ability to scavenge ROS and reduce MDA was partially abolished in the presence of ML385 (Figure 5D–G).

**Figure 6:**
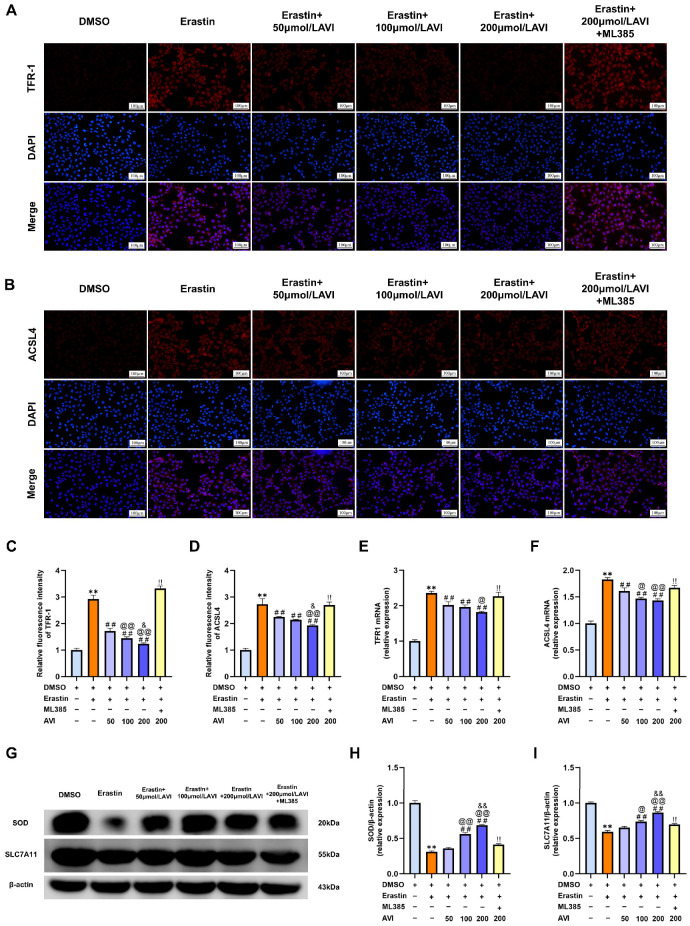
AVI modulates the expression of ferroptosis-related markers in HTR-8/SVneo cells. (**A**–**D**) IF staining of TFR-1 and ACSL4 distribution. Scale bar: 100 μm. (**E**,**F**) RT-qPCR analysis of TFR-1 and ACSL4 mRNA levels. (**G**) Western blotting analysis of SOD and SLC7A11 protein expression, with β-actin as the loading control. (**H**,**I**) Densitometric quantification of SOD and SLC7A11. Data are presented as mean ± SD (*n =* 3). ** *p* < 0.01 vs. Control group; ^##^ *p* < 0.01 vs. Model group; ^@^ *p* < 0.05, ^@@^ *p* < 0.01 vs. 50 μmol·L^−1^ AVI group; ^&^ *p* < 0.05, ^&&^ *p* < 0.01 vs. 100 μmol·L^−1^ AVI group; ^!!^ *p* < 0.01 vs. 200 μmol·L^−1^ AVI group.

Next, we evaluated the expression of established ferroptosis markers, utilizing IF and RT-qPCR to assess the localization and transcriptional levels of the pro-ferroptotic regulators TFR-1 and ACSL4. Both TFR-1 and ACSL4 were markedly upregulated in the Erastin-treated group, whereas AVI treatment effectively suppressed their expression. Conversely, ML385 administration antagonized the inhibitory effect of AVI on these markers (Figure 6A–F). Additionally, Erastin suppressed the protein expression of the crucial antioxidant mediators SOD and SLC7A11. While AVI effectively restored their expression, ML385 reversed this cytoprotection, impairing the antioxidant defense system (Figure 6G–I). In summary, AVI preserves mitochondrial function, mitigates oxidative damage and iron accumulation, and modulates the expression of core ferroptotic proteins to antagonize Erastin-induced ferroptosis in HTR-8/SVneo cells. Mechanistically, this robust protection is highly dependent on the NRF2 signaling pathway.

### 3.5. AVI Inhibits Ferroptosis in HTR-8/SVneo Cells via the KEAP1/NRF2/GPX4 Signaling Pathway

**Figure 7:**
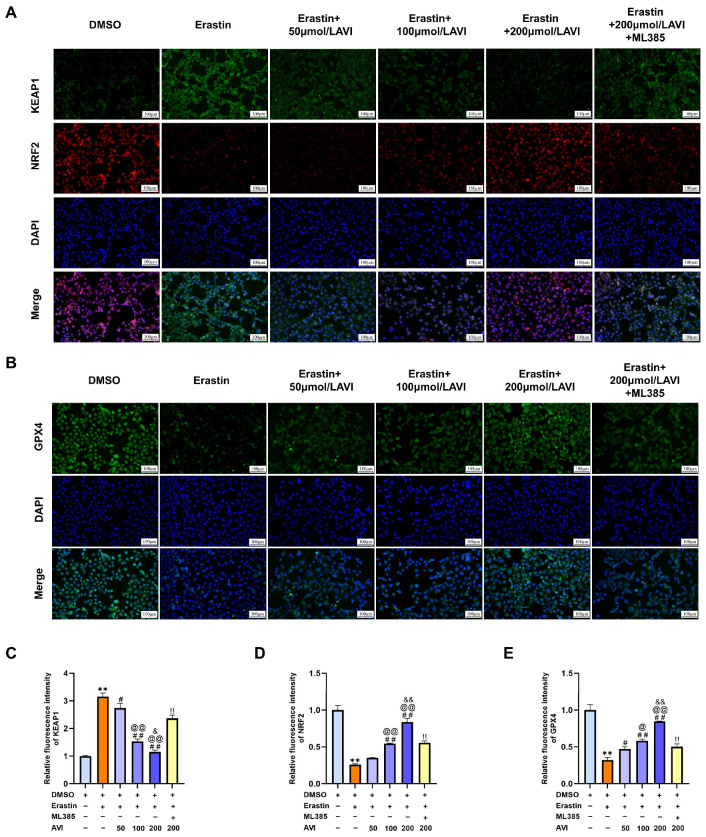
Fluorescence detection of ferroptosis in AVI-treated HTR-8/SVneo cells. (**A**–**E**) IF staining mapping the intracellular distribution of KEAP1, NRF2, and GPX4. Scale bar: 100 μm. Data are presented as mean ± SD (*n =* 3). ** *p* < 0.01 vs. Control group; ^#^ *p* < 0.05, ^##^ *p* < 0.01 vs. Model group; ^@^ *p* < 0.05, ^@@^ *p* < 0.01 vs. 50 μmol·L^−1^ AVI group; ^&^ *p* < 0.05, ^&&^ *p* < 0.01 vs. 100 μmol·L^−1^ AVI group; ^!!^ *p* < 0.01 vs. 200 μmol·L^−1^ AVI group.

**Figure 8:**
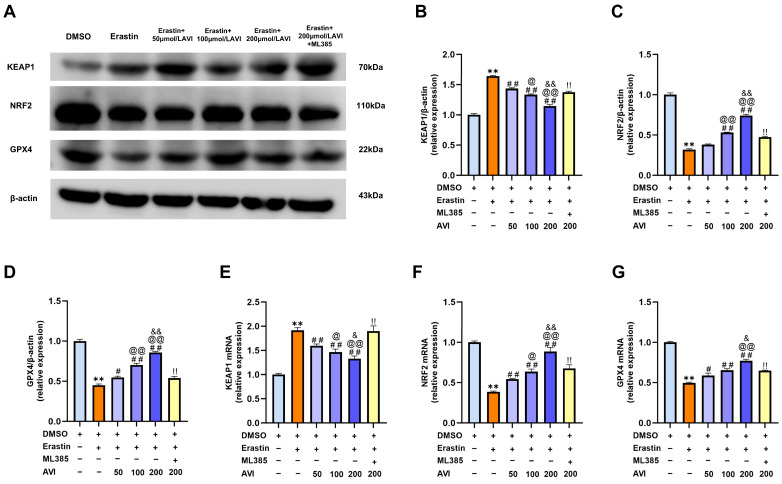
Western blotting analysis and RT-qPCR analysis of the KEAP1/NRF2/GPX4 signaling pathway. (**A**) Western blotting analysis of KEAP1, NRF2, and GPX4 protein expression. (**B**–**D**) Densitometric quantification of the respective proteins. (**E**–**G**) RT-qPCR analysis of KEAP1, NRF2, and GPX4 transcript levels. Data are presented as mean ± SD (*n =* 3). ** *p* < 0.01 vs. Control group; ^#^ *p* < 0.05, ^##^ *p* < 0.01 vs. Model group; ^@^ *p* < 0.05, ^@@^ *p* < 0.01 vs. 50 μmol·L^−1^ AVI group; ^&^ *p* < 0.05, ^&&^ *p* < 0.01 vs. 100 μmol·L^−1^ AVI group; ^!!^ *p* < 0.01 vs. 200 μmol·L^−1^ AVI group.

IF and Western blotting analyses revealed that, compared to the Erastin-treated group, AVI administration significantly attenuated KEAP1 protein levels, promoted NRF2 accumulation, and subsequently upregulated the expression of its downstream target, GPX4. Notably, co-treatment with the NRF2 inhibitor ML385 significantly abrogated these AVI-induced protein alterations, restoring KEAP1 levels while suppressing the upregulation of NRF2 and GPX4 (Figure 7A–E and Figure 8A–D). Consistent with these findings at the protein level, RT-qPCR analysis demonstrated that AVI reduced KEAP1 mRNA expression while concurrently elevating the transcript levels of NRF2 and GPX4. Furthermore, the addition of ML385 effectively reversed these AVI-mediated transcriptional changes (Figure 8E–G). Taken together, these data indicate that NRF2 inhibition effectively abolishes the AVI-induced activation of the KEAP1/NRF2/GPX4 axis. Ultimately, these findings establish that AVI confers protection against ferroptosis in HTR-8/SVneo cells through the activation of the KEAP1/NRF2/GPX4 signaling pathway.

## 4. Discussion

SA is a prevalent obstetric complication occurring prior to 20 weeks of gestation with a multifactorial etiology. Notably, the underlying pathogenesis remains elusive in approximately half of all clinical cases. Currently, clinical management primarily relies on supportive care. Although progesterone supplementation is frequently employed, specific preventive and therapeutic strategies targeting the fundamental pathophysiological processes of SA remain critically limited. Consequently, comprehensive elucidation of SA pathogenesis and subsequent development of targeted interventions hold substantial translational potential for improving pregnancy outcomes. Recently, the identification of novel fetal-protective agents derived from bioactive constituents of traditional Chinese medicine has emerged as a compelling research frontier. AVI, a principal triterpenoid saponin isolated from the roots of *Dipsacus* asperoides, exerts pleiotropic pharmacological effects, including potent anti-apoptotic and antioxidative properties, and shows substantial therapeutic potential for the prevention and treatment of pregnancy-related disorders such as threatened abortion, recurrent pregnancy loss, and preeclampsia.

The maintenance of normal gestation in rats relies critically on the structural integrity of the hemochorial placenta. Previous investigations utilizing rat models of SA have firmly established that region-specific lesions at the maternofetal interface constitute the fundamental pathological basis for embryo resorption [36]. Specifically, under pathological stress, the labyrinth zone, which is the primary hub for maternofetal exchange, frequently exhibits the collapse of its spongioform microvascular network, impaired maternal sinusoid remodeling, and extensive trophoblast death, which directly compromises fetal blood supply [37]. Concurrently, spongiotrophoblasts within the junctional zone typically undergo cellular dissociation and severe disorganization, while the maternal decidual zone is characterized by pronounced inflammatory cell infiltration and edema [38]. Highly consistent with these classic histopathological hallmarks, our H&E evaluation revealed comparable degenerative alterations in the placentas of SA rats. The model group demonstrated diminished microvascular spaces and widespread trophoblast necrosis within the labyrinth zone, alongside severe cellular disarray and pathological inflammation across the junctional and decidual zones. Notably, AVI administration effectively reversed these specific lesions; treatment successfully restored the labyrinthine spongioform vascular architecture, attenuated trophoblast necrosis, and resolved decidual inflammation. These prominent histological improvements confirm the protective efficacy of AVI against placental injury in SA, providing a robust foundation for investigating the underlying molecular mechanisms by which AVI improves placental function and pregnancy outcomes.

The potent antioxidant properties of AVI have been extensively validated in various pathological models. For instance, AVI has been shown to abrogate hypoxia-induced apoptosis in cardiomyocytes by activating the PI3K/Akt-CREB signaling pathway, thereby attenuating myocardial hypoxic injury [39,40]. Furthermore, AVI inhibits chondrocyte ferroptosis by activating the NRF2/GPX4/HO-1 signaling axis, alleviating oxidative stress-mediated mitochondrial dysfunction and endoplasmic reticulum stress, and thus suppressing chondrocyte apoptosis [41]. In the context of SA, the cytoprotective capacity of AVI against oxidative damage is equally prominent. Specifically, AVI protects endometrial stromal cells against oxidative stress-induced apoptosis by modulating the PI3K/Akt signaling pathway [42]. Additionally, AVI upregulates the expression of progesterone and its cognate receptor, dampens inflammatory responses in decidual cells, and facilitates early pregnancy decidualization via the Notch signaling pathway [43]. The present study aims to further elucidate the precise molecular mechanisms by which AVI targets trophoblasts for the management of SA. Here, we demonstrate that AVI regulates the KEAP1/NRF2/GPX4 axis in trophoblasts by activating the master antioxidant regulator NRF2, thereby inhibiting intracellular lipid peroxidation, blocking ferroptosis, and ultimately improving pregnancy outcomes.

Trophoblast ferroptosis constitutes a critical pathological event in the pathogenesis of SA. Clinical evidence indicates that aberrant trophoblast iron metabolism and accumulation of lipid peroxides are prevalent in the placental tissues of SA patients [34]. Hypoxia, inflammatory stress, and exposure to environmental pollutants can trigger placental ferroptosis via the activation of diverse signaling cascades, ultimately precipitating adverse pregnancy outcomes [44,45]. Building upon these observations, the present study elucidates the regulatory mechanisms through which AVI modulates this process. In vivo experiments demonstrated that AVI administration markedly modulated the expression of ferroptosis-associated markers in the placental tissues of SA model rats—specifically by downregulating the pro-ferroptotic factors TFR-1 and ACSL4, and restoring the expression of SLC7A11, while simultaneously reducing serum levels of Fe^2+^ and MDA. These findings substantiate the potent anti-ferroptotic efficacy of AVI in placental tissues.

The HTR-8/SVneo, an immortalized EVT-like model, is extensively utilized to investigate EVT dynamics [46]. By retaining the core phenotypic and functional characteristics of primary human EVTs, alongside robust viability and experimental reproducibility, this cell line reliably simulates in vivo EVT behavior. Thus, it serves as an ideal in vitro model for investigating the protective mechanisms of AVI against early gestational trophoblast dysfunction. The excessive intracellular accumulation of ROS during ferroptosis directly damages biological macromolecules, including proteins, lipids, and DNA. This oxidative damage extensively impairs trophoblast function, compromising cellular invasion and migration, and thereby disrupting normal placental development and physiological function [47,48]. Furthermore, ferroptosis is characteristically accompanied by ultrastructural and functional mitochondrial abnormalities, such as diminished mitochondrial cristae and depolarized membrane potential, which further exacerbate the disruption of cellular homeostasis [49]. Recent studies have demonstrated that hypoxia induces trophoblast ferroptosis via the HIF-1α/NCOA4 signaling pathway to precipitate SA [50], whereas nanoplastic exposure triggers syncytiotrophoblast ferroptosis, leading to placental senescence and fetal growth restriction [51]. In vitro assays further corroborated the direct cytoprotective effects of AVI on trophoblasts. Following Erastin exposure, the expression of antioxidant-related mediators (NRF2, GPX4, SLC7A11, and SOD) was significantly downregulated in HTR-8/SVneo cells, concomitant with the upregulation of KEAP1, TFR-1, and ACSL4. Crucially, AVI co-treatment dose-dependently reversed these aberrant molecular alterations, significantly restored mitochondrial function, and alleviated both oxidative damage and iron overload, thereby effectively antagonizing Erastin-induced trophoblast ferroptosis.

The NRF2 signaling pathway serves as a pivotal regulator in sustaining pregnancy homeostasis. As a master transcription factor within the cellular antioxidant defense system, NRF2 not only directly drives the transcription of downstream cytoprotective genes but also forms a sophisticated regulatory feedback loop through interactions with its primary negative regulator, KEAP1, ensuring precise functional execution. Recent studies have demonstrated that pharmacological inhibition of NRF2 using ML385 significantly downregulates the expression of both NRF2 and KEAP1 at the transcript and protein levels in human primary cells [52,53]. In the context of pregnancy, targeted inhibition of NRF2 in trophoblasts suppresses heme HO-1 expression, thereby precipitating SA [54], suggesting that physiological NRF2 activation constitutes an indispensable safeguard against oxidative stress-induced injury during gestation. Clinically, NRF2 protein expression is markedly diminished in the placental tissues of patients with preeclampsia, associated with exacerbated mitochondrial dysfunction and severe oxidative damage in trophoblasts [55,56]. Collectively, these findings underscore that the robust activation of NRF2 signaling constitutes an indispensable safeguard against oxidative stress-induced gestational injury. Furthermore, in silico molecular simulation data provide compelling preliminary evidence that AVI directly targets NRF2. Molecular docking analyses revealed that AVI exhibits high binding affinities for key ferroptosis-associated targets, including KEAP1, NRF2, GPX4, SLC7A11, and TFR-1, among which its binding stability with NRF2 is markedly superior. Subsequent molecular dynamics simulations confirmed that within the KEAP1/NRF2/GPX4 signaling axis, AVI forms the most stable conformation with the NRF2 protein during extended simulation trajectories, identifying NRF2 as a primary therapeutic target for AVI in the treatment of SA. Biologically, co-treatment of trophoblasts with the NRF2 inhibitor ML385 significantly abrogated the cytoprotective efficacy of AVI, confirming an NRF2-dependent mechanism of action. Consistent with previous reports showing that NRF2 activation mitigates ROS accumulation, suppresses lipid peroxidation, and reduces embryo resorption in SA mouse models [55,57]. Furthermore, dysregulation of the KEAP1/NRF2 regulatory axis during gestation impairs redox homeostasis, culminating in adverse pregnancy outcomes [58]. In our in vivo SA model, KEAP1 expression was significantly elevated in placental tissues, concomitant with the marked downregulation of NRF2 and its downstream targets, GPX4 and SLC7A11. Crucially, AVI administration effectively reversed these pathological aberrations. Taken together, these findings elucidate that AVI mitigates SA by directly activating NRF2 and modulating the KEAP1/NRF2/GPX4 axis, thereby preserving trophoblast function and sustaining pregnancy homeostasis.

While the present study elucidates the mechanisms by which AVI protects trophoblasts and mitigates SA through the activation of NRF2 and subsequent upregulation of GPX4, several limitations warrant consideration for future research. Firstly, the in vivo experiments utilized rat models, which possess inherent structural and physiological divergences from human placentas. Consequently, these models cannot fully recapitulate the pathological hallmarks of clinical SA, thereby limiting the direct translational applicability of our findings. Furthermore, because this investigation primarily focuses on the molecular regulation of trophoblast ferroptosis by AVI, the exploration of placental histopathological lesions remains limited in scope. Our assessment was restricted to qualitative morphological observations of regional placental damage, lacking systematic quantitative analyses and in-depth pathological verification of zonal lesion disparities. Additionally, our mechanistic validation relied predominantly on the pharmacological inhibitor ML385; future studies should corroborate these findings utilizing trophoblast-specific NRF2 knockout models both in vitro and in vivo. Subsequently, inherent discrepancies exist between current experimental models and the complex human in vivo microenvironment. To more accurately recapitulate clinical conditions, future investigations should validate the therapeutic efficacy of AVI using primary human trophoblasts or advanced placental organoid systems. Furthermore, the intricate downstream regulatory networks of NRF2 and the potential crosstalk between ferroptosis and alternative programmed cell death pathways (e.g., apoptosis and pyroptosis) remain incompletely elucidated, and subsequent research employing multi-omics technologies is required for in-depth exploration of these mechanisms. Ultimately, as our study was mainly performed in isolated trophoblasts, the impact of AVI on the broader multicellular crosstalk at the maternal-fetal interface remains unknown. Future studies employing co-culture systems, comprehensive organoid models, or single-cell RNA sequencing are imperative to fully elucidate these complex intercellular dynamics.

## 5. Conclusions

In conclusion, the present study demonstrates that AVI effectively attenuates Erastin-induced ferroptosis in trophoblasts by modulating the KEAP1/NRF2/GPX4 signaling axis. These findings further elucidate the role of ferroptosis and trophoblast dysfunction in the pathogenesis of SA. Furthermore, this work provides robust experimental evidence supporting the therapeutic potential of AVI in managing SA through the targeted inhibition of trophoblast ferroptosis.

## Figures and Tables

**Table 1:** Primers for Real-Time qPCR.

Genes	Accession No.	Primer Sequences (5′-3′)	Product Length (bp)
*β-Actin*	NM_001101.5	F: GAGAAAATCTGGCACCACACC	177
		R: GGATAGCACAGCCTGGATAGCAA	
*KEAP1*	NM_012289.4	F: GCTGTCCTCAATCGTCTCCTT	104
		R: ATCATTCGCCACTCGTTCCT	
*NRF2*	NM_006164.5	F: ATGCCACAGTCAACACAGATT	126
		R: GCCCATTTAGAAGTTCAGAGAGT	
*GPX4*	NM_002085.5	F: GAGGCAAGACCGAAGTAAACTAC	100
		R: CCGAACTGGTTACACGGGAA	
*ACSL4*	NM_004458.3	F: ATCCTAATTOCTCGACAGAACATC	190
		R: ACTCTCCTOCTTGTAACTTCACTA	
*TFR-1*	NM_003234.4	F: TGAGAGGTACAACAGCCAACT	130
		R: GGAAGTAGCACGGAAGAAGTC	

**Table 2:** Comparative molecular dynamics simulation parameters for proteins within the KEAP1/NRF2/GPX4 signaling axis.

Key Indicators	KEAP1	NRF2	GPX4
MMPBSA binding free energy (kJ/mol)	−9.47	−12.31	−8.57
Stable binding stage of complex	None	After 85 ns	After 75 ns
Key binding amino acids	HISPRO1, HIS195	ARG326, ARG470, THR609	ARG64, ASP96, LYS26&94, HIS55
Main interactions	None	Van der waals forces	Van der waals forces
Number of stable hydrogen bonds	None	1–3	1
Protein free energy landscape characteristics	Non-single stable structure	Single stable structure	Single stable structure

## Data Availability

The original contributions presented in this study are included in the article. Further inquiries can be directed to the corresponding authors.

## Abbreviations

The following abbreviations are used in this manuscript:

ACSL4	Acyl-CoA Synthetase Long-chain family member 4
AVI	Asperosaponin VI
BCA	Bicinchoninic acid
CCK-8	Cell Counting Kit-8
CT	Cytotrophoblast
DCF	Dichlorofluorescein
DCFH-DA	Dichlorodihydrofluorescein diacetate
Fe^2+^	Ferrous iron
GPX4	Glutathione peroxidase 4
GSH-Px	Glutathione peroxidase
H&E	Hematoxylin and eosin
HRP	Horseradish Peroxidase
IF	Immunofluorescence
KEAP1	Kelch-like ECH-associated protein 1
MDA	Malondialdehyde
MD	Molecular dynamic
MFI	Mean fluorescence intensity
MMP	Mitochondrial membrane potential
NRF2	Nuclear factor erythroid 2-related factor 2
PBS	Phosphate-buffered Saline
PFA	Paraformaldehyde
Rg	Radius of gyration
RMSD	Root mean square deviation
RMSF	Root mean square fluctuation
ROS	Reactive oxygen species
RT-qPCR	Reverse transcription-quantitative polymerase chain reaction
SA	Spontaneous abortion
SLC7A11	Solute carrier family 7 member 11
SOD	Superoxide dismutase
TEM	Transmission electron microscopy
TFR-1	Transferrin receptor 1

## Appendix A

### Appendix A.1. MD Simulation of the KEAP1-AVI Complex

While the RMSD of the KEAP1 apoprotein remained stable (<0.2 nm), the RMSD of the KEAP1-AVI complex exhibited pronounced fluctuations, particularly during the 80–100 ns interval (Figure A1A), indicating a lack of stable ligand binding. However, the magnitude of the ligand RMSD fluctuation was lower than that observed in the GPX4 group. RMSF analysis revealed significant flexibility near residues 60, 125, 175, and 200, suggesting these as potential transient binding sites for the ligand (Figure A1B). Both the Rg (Figure A1C) and the SASA (Figure A1D) remained relatively stable, indicating that the overall protein architecture did not undergo substantial conformational changes. Hydrogen bond analysis demonstrated the presence of 1–2 intermolecular hydrogen bonds during the initial 0–50 ns, which transiently increased to 2–4 bonds between 50 and 80 ns; however, hydrogen bonding was nearly abolished during the final 80–100 ns interval (Figure A1E). Similarly, interaction energy analysis revealed enhanced electrostatic interactions during the 50–80 ns phase, which subsequently dissipated in the later stages of the simulation (Figure A1F). The MM/PBSA binding free energy was calculated at −9.47 kJ/mol, representing a marginally more favorable binding affinity compared to the GPX4 complex (Figure A1G). Per-residue free energy decomposition analysis identified Pro1 and His195 as key contributors to the binding interaction (Figure A1H). Finally, the free energy landscape (FEL) displayed a broad distribution of energy minima, further suggesting that the protein sampled multiple conformational states throughout the simulation (Figure A1I).

### Appendix A.2. Molecular Dynamics Simulation of the GPX4-AVI Complex

RMSD analysis revealed that the GPX4 apoprotein maintained structural stability throughout the simulation, with an RMSD of <0.2 nm (Figure A2A). In contrast, the RMSD of the GPX4-AVI complex exhibited minor fluctuations from 0 to 55 ns, followed by pronounced variations between 55 and 75 ns, before stabilizing after 75 ns. This trajectory suggests that the ligand established stable interactions with the protein only during the latter stages of the simulation. Furthermore, the intrinsic RMSD of the ligand continued to fluctuate beyond 45 ns, implying a potentially dynamic or partial binding mode. RMSF analysis demonstrated that the flexibility of most GPX4 residues remained below 0.2 nm, indicative of substantial structural rigidity (Figure A2B). Both the Rg and the SASA remained consistent, confirming the absence of significant conformational changes in the protein architecture (Figure A2C,D). Hydrogen bond analysis identified 1 to 4 intermolecular hydrogen bonds during the initial 0–50 ns interval, which stabilized to approximately a single hydrogen bond during the final 80–100 ns phase (Figure A2E). Interaction energy assessments indicated that van der Waals forces predominated during the stable binding phase (Figure A2F). The MM/PBSA-derived binding free energy was calculated at −8.57 kJ/mol, reflecting a relatively weak binding affinity (Figure A2G). Per-residue free energy decomposition analysis revealed that Arg64, Asp96, Lys26, Lys94, and His55 were key contributors to the binding interaction (Figure A2H). Finally, the FEL displayed a single prominent energy minimum, further corroborating the conformational stability of the protein (Figure A2I).
